# A novel yeast model detects Nrf2 and Keap1 interactions with Hsp90

**DOI:** 10.1242/dmm.049258

**Published:** 2022-04-13

**Authors:** Vy Ngo, Anne Brickenden, Hansen Liu, Cynthia Yeung, Wing-Yiu Choy, Martin L. Duennwald

**Affiliations:** 1 Department of Pathology and Laboratory Medicine, Schulich School of Medicine and Dentistry, University of Western Ontario, London, ON N6A 3K7, Canada; 2 Department of Biochemistry, Schulich School of Medicine and Dentistry, University of Western Ontario, London, ON N6A 3K7, Canada; 3 Department of Anatomy and Cell Biology, Schulich School of Medicine and Dentistry, University of Western Ontario, London, ON N6A 3K7, Canada

**Keywords:** Nrf2, Keap1, Hsp90, Molecular chaperones, Protein interactions, Yeast model

## Abstract

Nrf2 is the master transcriptional regulator of cellular responses against oxidative stress. It is chiefly regulated by Keap1, a substrate adaptor protein that mediates Nrf2 degradation. Nrf2 activity is also influenced by many other protein interactions that provide Keap1-independent regulation. To study Nrf2 regulation, we established and characterized yeast models expressing human Nrf2 (also known as NFE2L2), Keap1 and other proteins that interact with and regulate Nrf2. Yeast models have been well established as powerful tools to study protein function and genetic and physical protein-protein interactions. In this work, we recapitulate previously described Nrf2 interactions in yeast and discover that Nrf2 interacts with the molecular chaperone Hsp90. Our work establishes yeast as a useful tool to study Nrf2 interactions and provides new insight into the crosstalk between the antioxidant response and the heat shock response.

## Abstract (precis)

**Summary:** We studied the interactions of human Nrf2 in a novel budding yeast model. We recapitulate previously described Nrf2 interactions and discover that Nrf2 interacts with Hsp90, establishing yeast as a useful tool to study Nrf2 interactions.

## INTRODUCTION

Nuclear factor erythroid 2-related factor 2 (Nrf2; also known as NFE2L2) is the master transcriptional regulator of cellular responses against oxidative stress (Moi et al., 1994). Nrf2 is negatively regulated by Kelch-like ECH-associated protein (Keap1), a substrate adaptor protein that binds to Nrf2 in the cytoplasm to promote Nrf2 ubiquitination via the cullin 3 (Cul3) E3 ubiquitin ligase for proteasomal degradation under basal conditions (Itoh et al., 1999, 2003; McMahon et al., 2003; Nguyen et al., 2003; Kobayashi et al., 2004, 2006). Under oxidative stress conditions, specific stress-sensing cysteine residues in Keap1 are modified (Dinkova-Kostova et al., 2002; Zhang and Hannink, 2003; Wakabayashi et al., 2004), leading to a conformational change that impairs the interaction between Keap1 and Nrf2, thereby preventing Nrf2 ubiquitination and degradation and stabilizing Nrf2 for accumulation, nuclear translocation and the induction of the expression of cytoprotective antioxidant genes (Itoh et al., 1999, 2003; McMahon et al., 2003; Kobayashi et al., 2006).

Stability, and therefore the activity of Nrf2, is tightly regulated by two binding events to Keap1: Keap1 first recruits Nrf2 by binding to the high-affinity ETGE motif within the Neh2 domain of Nrf2, and subsequent binding at the low-affinity DLG motif within Neh2 locks Nrf2 in place by orienting the lysine residues within Neh2 in the correct position for ubiquitination (Tong et al., 2006, 2007). This two-site binding model has been widely accepted as the primary mechanism of Keap1-mediated Nrf2 regulation. Mutations that disrupt the Keap1-Nrf2 interaction alter Nrf2 regulation and contribute to the pathogenesis of many human diseases (Padmanabhan et al., 2006; Shibata et al., 2008). For example, gain-of-function mutations within the Keap1-binding domain of Nrf2, specifically within the DLG motif (e.g. L30F) and ETGE motif (e.g. T80R), impair its recognition by Keap1-Cul3, leading to the dysregulation and subsequent hyperactivation of Nrf2 in lung cancer (Shibata et al., 2008). In addition to Keap1, Nrf2 regulation is greatly dependent on its interactions with many other proteins.

An interesting alternative mechanism of Nrf2 regulation first proposed by Zhang et al. identified p21 (also known as p21^WAF1/Cip1^ or CDKN1A) as a regulator of Nrf2 transcriptional activity (Chen et al., 2009). p21 is a cyclin-dependent kinase inhibitor with well-established roles in p53-regulated tumor suppression, including cell cycle control, DNA replication and repair, and apoptosis (Xiong et al., 1993; Gartel and Radhakrishnan, 2005; Abbas and Dutta, 2009). The authors found that ablation of p21 results in increased cellular levels of reactive oxygen species (ROS). Reciprocal immunoprecipitation assays and pull-down experiments for p21 and Nrf2 suggest that p21 directly interacts with Nrf2 by competing with Keap1 for binding, indicating that p21 binding to Nrf2 prevents Keap1-directed Nrf2 degradation (Chen et al., 2009). Several studies have linked the overexpression of cytoplasmic p21 to decreased responsiveness to chemotherapy and radiotherapy (Liu et al., 2003) and poor prognosis in numerous cancers (Baretton et al., 1999; Bae et al., 2001; Cheung et al., 2001). The cellular and molecular mechanisms of the interaction between p21 and Nrf2 remain unclear.

Other key proteins in the Nrf2 interactome that were investigated in this study include β-transducin repeat-containing protein (βTrCP; also known as FBXW11), which acts as a substrate receptor for the Skp1-Cul1-Rbx1/Roc1 ubiquitin ligase complex involved in Keap1-independent Nrf2 degradation (Rada et al., 2011; Chowdhry et al., 2013); Cul3, which binds Keap1 in the cytosol and, upon Keap1-Nrf2 binding, polyubiquitinates Nrf2 for degradation by the 26S proteasome (Kobayashi et al., 2004; Zhang et al., 2004); and the underexplored prothymosin alpha (ProTα; also known as PTMA), which is thought to inhibit the Keap1-Nrf2 complex by competing with Nrf2 for Keap1 binding (Karapetian et al., 2005; Khan et al., 2013).

In this work, we established a novel approach for studying the interactions of human Nrf2 in the budding yeast *Saccharomyces cerevisiae*. Yeast and human cells share fundamental commonalities in many conserved cellular processes, making yeast a powerful model system for studying the mechanisms of important cellular processes, including those that underlie protein regulation and human disease (Hartwell, 2005; Smith and Snyder, 2006; Tenreiro and Outeiro, 2010; Di Gregorio and Duennwald, 2018). Yeast is a formidable model to identify and characterize genetic interactions, which can be defined as the phenomenon by which the phenotypic effects of the function of one gene modify the phenotypic effects of another gene or genes (Mani et al., 2008). Yeast can also be used to study physical protein-protein interactions (PPIs) by employing the yeast two-hybrid system (Fields and Song, 1989) or split-ubiquitin system (Johnsson and Varshavsky, 1994) to identify and characterize such physical interactions that occur through specific contact and molecular docking (De Las Rivas and Fontanillo, 2010). Yeast does not express any close Nrf2 homolog, which allows us to minimize interference with endogenous Nrf2 regulators as occurs in mammalian cells. This feature makes yeast an optimal living test tube for studying Nrf2 interactions without the interference of Nrf2 transcriptional activity. Our yeast model of human Nrf2 confirmed previously established genetic and physical Nrf2 interactions and allowed us to characterize a previously unexplored interaction between Nrf2 and the molecular chaperone heat shock protein 90 (Hsp90).

## RESULTS

### Expression of human Nrf2 and associated proteins in yeast

**Fig. 1.**
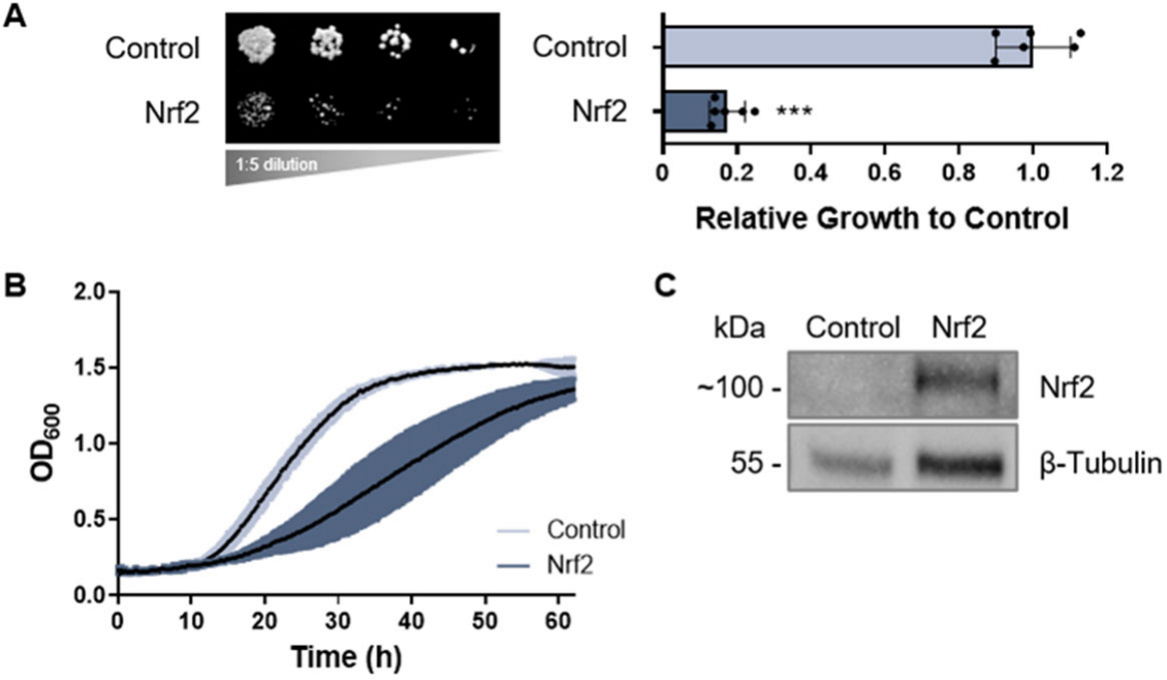
**Expression of human Nrf2 in yeast.** (A) Growth assay of yeast cells expressing human Nrf2 (left). Relative growth is quantified to the right of the image. Human Nrf2 is toxic in yeast (*P*<0.001). Means derived from five biological replicates were used during analysis. Means were analyzed using a two-tailed unpaired Student's *t*-test. Data are expressed as mean±s.d. ***P<0.001. (B) Growth curve of yeast cells expressing Nrf2 grown in liquid culture. Means derived from three biological replicates were obtained. Data are expressed as mean±s.d. (C) Nrf2 protein expression in yeast documented by western blot analysis, with β-tubulin serving as the internal loading control.

Yeast growth assays were used to assess the relative growth and toxicity of select human proteins within the Nrf2 interactome expressed in yeast to determine genetic interactions with Nrf2, including human Keap1, p21, βTrCP, Cul3 and ProTα. Human Nrf2 expressed in yeast led to toxicity, defined as an impaired growth phenotype on growth media compared to the empty vector control (Fig. 1A, left). Relative growth on solid media was quantified to show statistical significance, performed as described previously (Petropavlovskiy et al., 2020) (Fig. 1A, right). Means derived from five biological replicates were used for these analyses. Our results were also confirmed quantitatively by assessing the growth rate of yeast cells grown in liquid culture (Fig. 1B). Nrf2 toxicity in yeast is likely attributed to cellular quiescence but not cell death, as determined by a propidium iodide (PI) assay, which showed no cell death in yeast cells expressing Nrf2 compared to the boiled positive control for cell death (Fig. S1). Protein expression of Nrf2 in yeast was confirmed by western blot analysis (Fig. 1C). Fluorescence microscopy also confirmed protein expression through the visualization of a yellow fluorescent protein (YFP) or Discosoma red fluorescent protein (DsRed) tag fused to the carboxy-terminus of Nrf2, which shows that Nrf2 is diffusely localized in the yeast cytoplasm and nucleus (Fig. S2A). In ensuing studies, we exploited Nrf2 toxicity as a tractable phenotype for our genetic interaction studies, as done previously in well-established yeast models expressing other human proteins (Hartwell, 2005; Tenreiro and Outeiro, 2010; Di Gregorio and Duennwald, 2018).

**Fig. 2.**
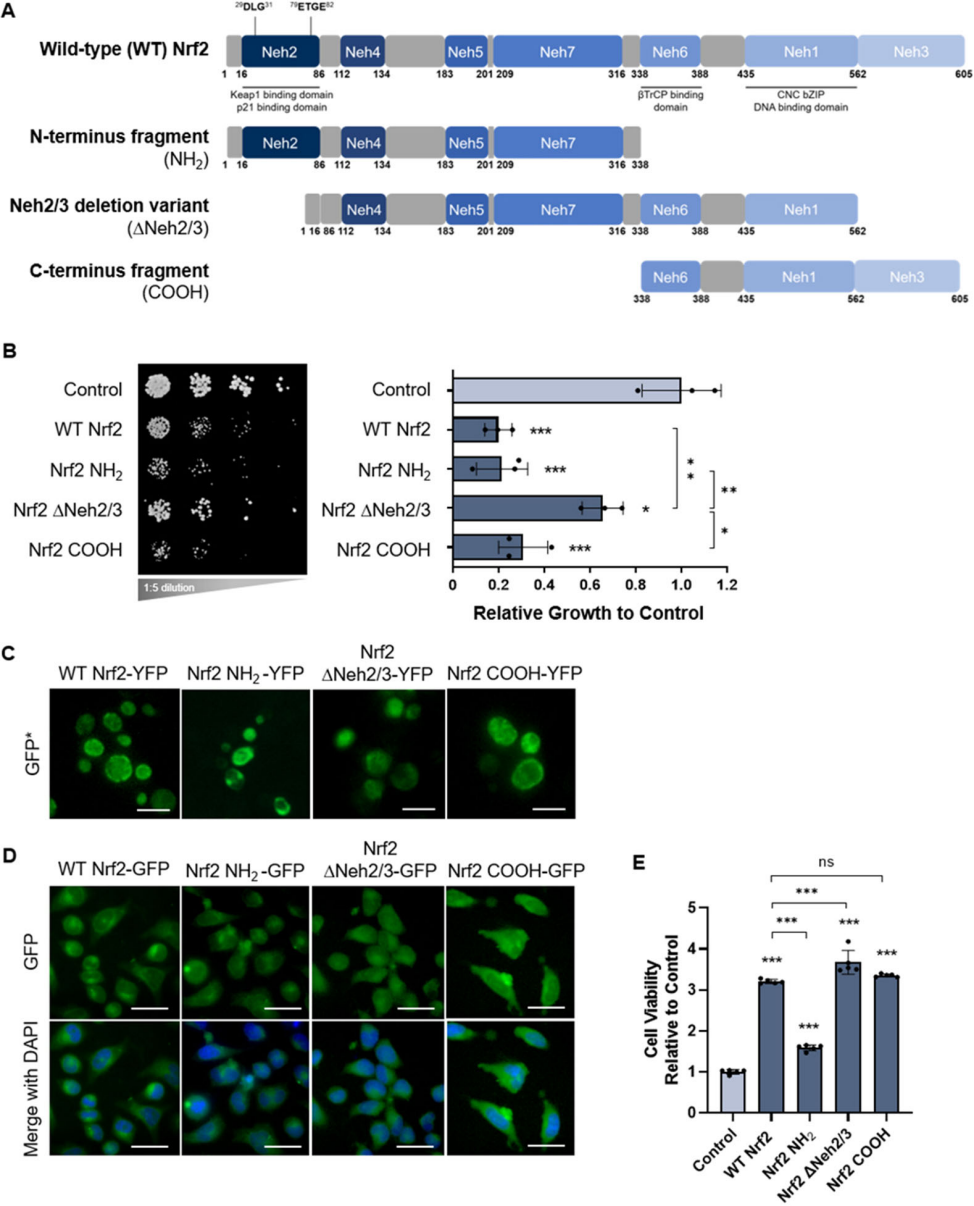
**Nrf2 fragments expressed in yeast and human cells.** (A) Schematic representation of the domains within full-length wild-type human Nrf2 and the fragmented variants of Nrf2: the N-terminal fragment (NH_2_), the Neh2/3 deletion variant (ΔNeh2/3) and the C-terminal fragment (COOH). (B) Growth assay of yeast cells expressing the three Nrf2 fragments. (C) Fluorescence microscopy of yeast cells expressing the YFP-tagged Nrf2 fragments. Scale bars: 10 μm. (D) Fluorescence microscopy of HeLa cells expressing the Nrf2 GFP-tagged fragments (top row), merged with DAPI nuclear staining (bottom row). Scale bars: 25 μm. (E) Relative viability of HeLa cells transfected with wild-type Nrf2 and its fragments. In B and E, means derived from a minimum of three biological replicates were used during analysis. Means were analyzed using one-way ANOVA followed by Tukey's post hoc test. Data are expressed as mean±s.d. ns, not significant; *P<0.05, **P<0.01, ***P<0.001.

To assess which regions in Nrf2 gave rise to the protein's toxic phenotype in yeast, three fragments of Nrf2 were examined (Fig. 2A): (1) the N-terminal (NH_2_) fragment, consisting of the Neh2, Neh4, Neh5 and Neh7 domains; (2) the ΔNeh2/3 variant, with deletions of the Neh2 and Neh3 domains; and (3) the C-terminal (COOH) fragment, containing the Neh6, Neh1, and Neh3 domains. Like wild-type Nrf2, the NH_2_ and COOH fragments were toxic in yeast, whereas the ΔNeh2/3 variant was not (Fig. 2B). Fluorescence microscopy showed diffuse cytoplasmic localization of ΔNeh2/3-YFP but greater localization to fluorescent foci for Nrf2 NH_2_-YFP_­_ and Nrf2 COOH-YFP, which might indicate the formation of protein inclusions, particularly for the NH_2_ fragment (Fig. 2C). The NH_2_ fragment contains the crucial Neh2 (Keap1-binding) domain of Nrf2, which has been characterized as intrinsically disordered (Tong et al., 2006). Intrinsically disordered proteins or regions are more prone to misfolding under certain conditions (Uversky, 2011); thus, the Neh2 domain is plausibly a driver of misfolding and possibly the ensuing toxicity in yeast.

GFP-tagged protein constructs for mammalian expression in HeLa cells recapitulated the fluorescence microscopy results observed in yeast, illustrating the formation of protein inclusions for Nrf2 NH_2_-GFP (Fig. 2D). Upon analyzing the cell viability of wild-type Nrf2 and its fragmented variants in HeLa cells, we found that overexpression of wild-type Nrf2 is not toxic to HeLa cells, but instead promoted increased cell viability [determined by the quantification of ATP levels, which indicates the presence of metabolically active cells (Crouch et al., 1993)] compared to the untreated control; however, cell viability was impaired with the expression of Nrf2 NH_2_ (Fig. 2E). Experiments in the HEK293 cell line reproduced the results observed in HeLa cells (Fig. S3).

**Fig. 3.**
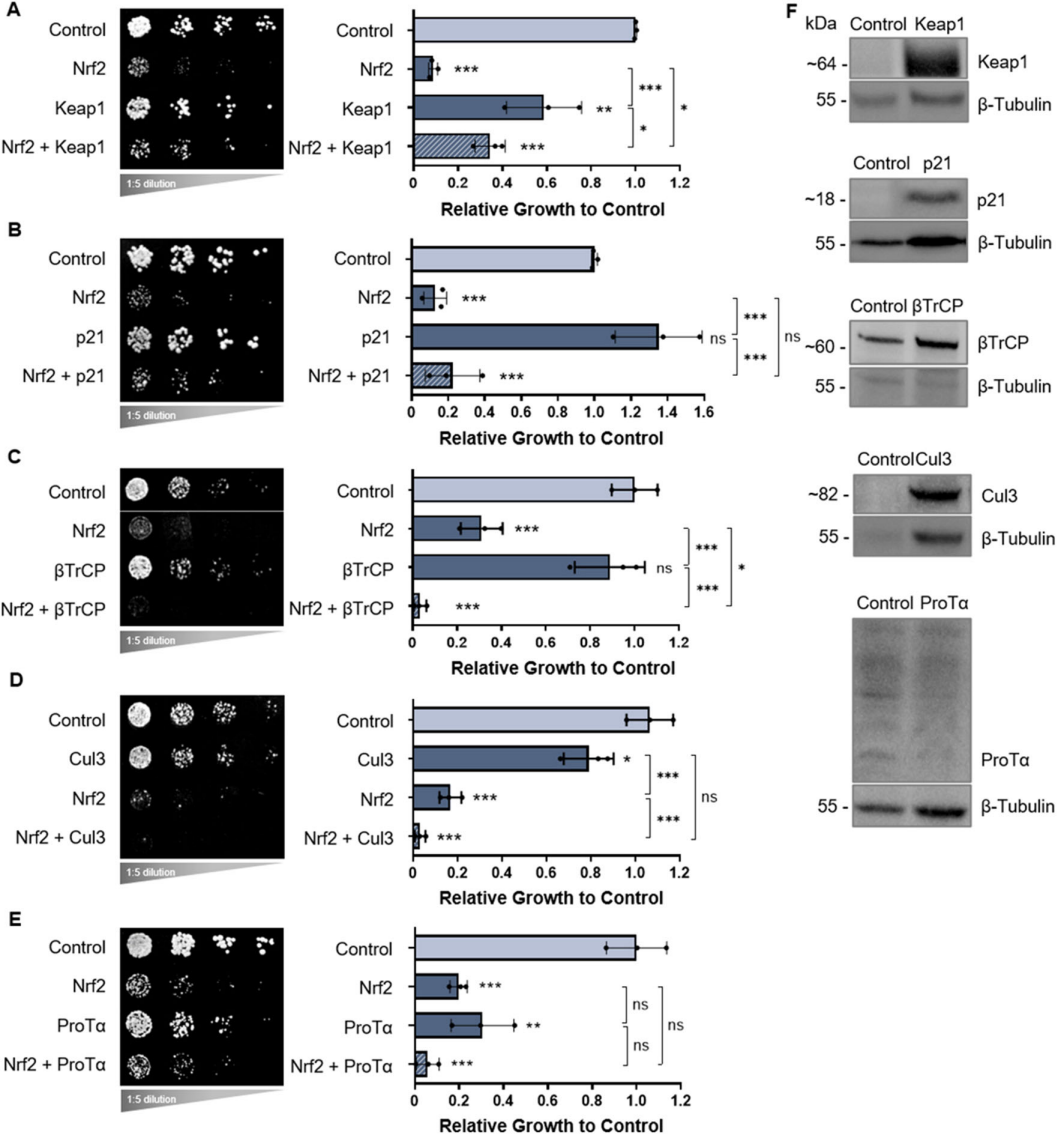
**Co-expression of Nrf2 with other Nrf2-associated proteins.** (A-E) Growth assays of yeast cells co-expressing human Nrf2 along with the following human proteins within the Nrf2 interactome: Keap1 (A), p21 (B), βTrCP (C), Cul3 (D) and ProTα (E). All growth assays are quantified as shown on the right with means derived from three biological replicates. Means were analyzed using one-way ANOVA followed by Tukey's post hoc test. Data are expressed as mean±s.d. ns, not significant; *P<0.05, **P<0.01, ***P<0.001. (F) Keap1, p21, βTrCP and Cul3 expression are shown by western blot analysis, with β-tubulin serving as the internal loading control. ProTα could not be reliably detected (see text for details).

To assess genetic Nrf2 interactions, we co-transformed yeast with well-established Nrf2-interacting proteins described in the literature, including Keap1 (Itoh et al., 1999), p21 (Chen et al., 2009) and βTrCP (Rada et al., 2011; Chowdhry et al., 2013), as well as the Keap1-interacting Cul3 (Kobayashi et al., 2004; Zhang et al., 2004) and ProTα (Karapetian et al., 2005) to serve as negative controls. Keap1 expression in yeast was only mildly toxic. Keap1 co-expression with Nrf2 improved growth relative to the expression of Nrf2 alone (Fig. 3A), i.e. the expression of Keap1 resulted in a partial rescue of Nrf2 toxicity, suggesting a genetic interaction. This was also observed in liquid growth assays (Fig. S4). Thus, our yeast model confirmed the well-established canonical genetic interaction between Keap1 and Nrf2 in mammalian cells.

When human p21 was co-expressed with Nrf2, there was no significant rescue of Nrf2 toxicity, indicating no detectable genetic p21-Nrf2 interaction in yeast (Fig. 3B), although a physical PPI may still exist (to be discussed). Co-expression with human βTrCP exacerbated Nrf2 toxicity, indicating a βTrCP-Nrf2 genetic interaction (Fig. 3C) as suggested before (Rada et al., 2011; Chowdhry et al., 2013). Of note, this may be a result of synthetic toxicity and thus a limitation of the yeast model because the kinase GSK3 is not expressed in yeast – the GSK3-dependent phosphorylation of Nrf2 is a prerequisite for the interaction of Nrf2 with βTrCP (Rada et al., 2011; Chowdhry et al., 2013). Moreover, co-expression of Nrf2 with human Cul3 did not produce detectable changes to the toxic Nrf2 phenotype (Fig. 3D), nor did co-expression with human ProTα (Fig. 3E). This is consistent with literature describing both Cul3 and ProTα as binding partners of Keap1 (Karapetian et al., 2005; Khan et al., 2013) but not Nrf2. Protein expression in yeast was confirmed by western blot analysis for all proteins of interest (Fig. 3F), except for ProTα, for which no commercially available yeast-compatible antibody could be found. Fluorescence microscopy with YFP-tagged constructs also confirmed expression in yeast for all proteins of interest, both alone and co-expressed with Nrf2 (Fig. S2B). Given the resolution of the fluorescent images, no more specific localization information could be confidently deduced.

**Fig. 4.**
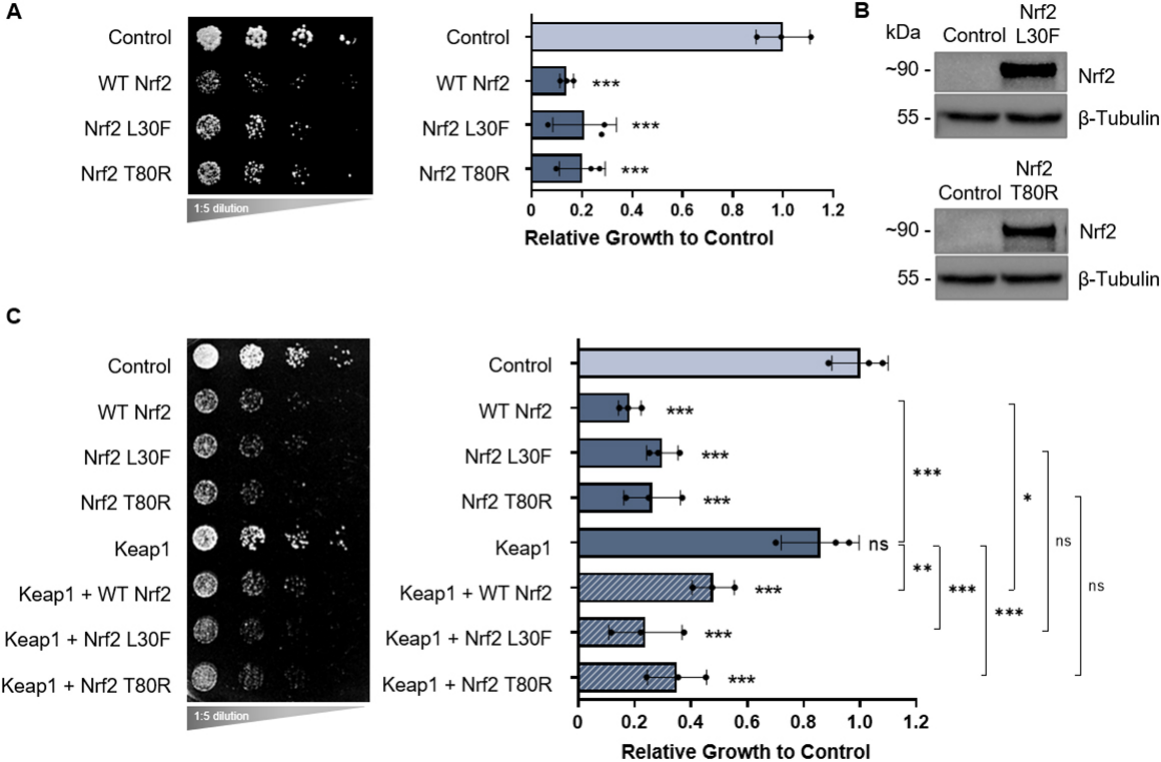
**Nrf2 mutant variants expressed in yeast.** (A) Growth assays of yeast cells expressing wild-type human Nrf2 and its mutant variants, L30F and T80R. (B) Expression of Nrf2 L30F and Nrf2 T80R in yeast documented by western blot analysis, with β-tubulin serving as the internal loading control. (C) Growth assays of yeast cells expressing wild-type Nrf2 and its mutant variants co-expressed with Keap1. In A and C, means derived from three biological replicates were used during analysis. Means were analyzed using one-way ANOVA followed by Tukey's post hoc test. Data are expressed as mean±s.d. ns, not significant; *P<0.05, **P<0.01, ***P<0.001.

In addition to wild-type Nrf2, we examined two variants of Nrf2 with mutations in the Keap1-binding Neh2 domain of Nrf2: an L30F mutation in the DLG motif and a T80R mutation in the ETGE motif. Like wild-type Nrf2, Nrf2 L30F and T80R were both toxic in yeast (Fig. 4A). Protein expression was confirmed by western blot analysis (Fig. 4B) and fluorescence microscopy (Fig. S2C), which showed no major differences between wild-type and mutant variant Nrf2 steady-state protein levels and subcellular localization. We then assessed the genetic interactions of the L30F and T80R variants. When Keap1 was co-expressed with Nrf2 L30F or T80R, the ability of Keap1 to rescue Nrf2 toxicity (observed in Fig. 3A) was impaired (Fig. 4C). As observed for wild-type Nrf2, co-expression of p21 with the L30F and T80R variants also did not affect Nrf2 toxicity (Fig. S5; refer to Figs S7-S9 for the technical control plates used in all yeast expression studies). Collectively, these experiments demonstrate how simple growth assays of yeast cells expressing wild-type Nrf2, fragmented and mutated variants of Nrf2, and Nrf2-associated proteins allow for the assessment of genetic interactions, such as that with Keap1 and βTrCP, that regulate Nrf2 in mammalian cells.

### The split-ubiquitin system detects PPIs of Nrf2

**Fig. 5.**
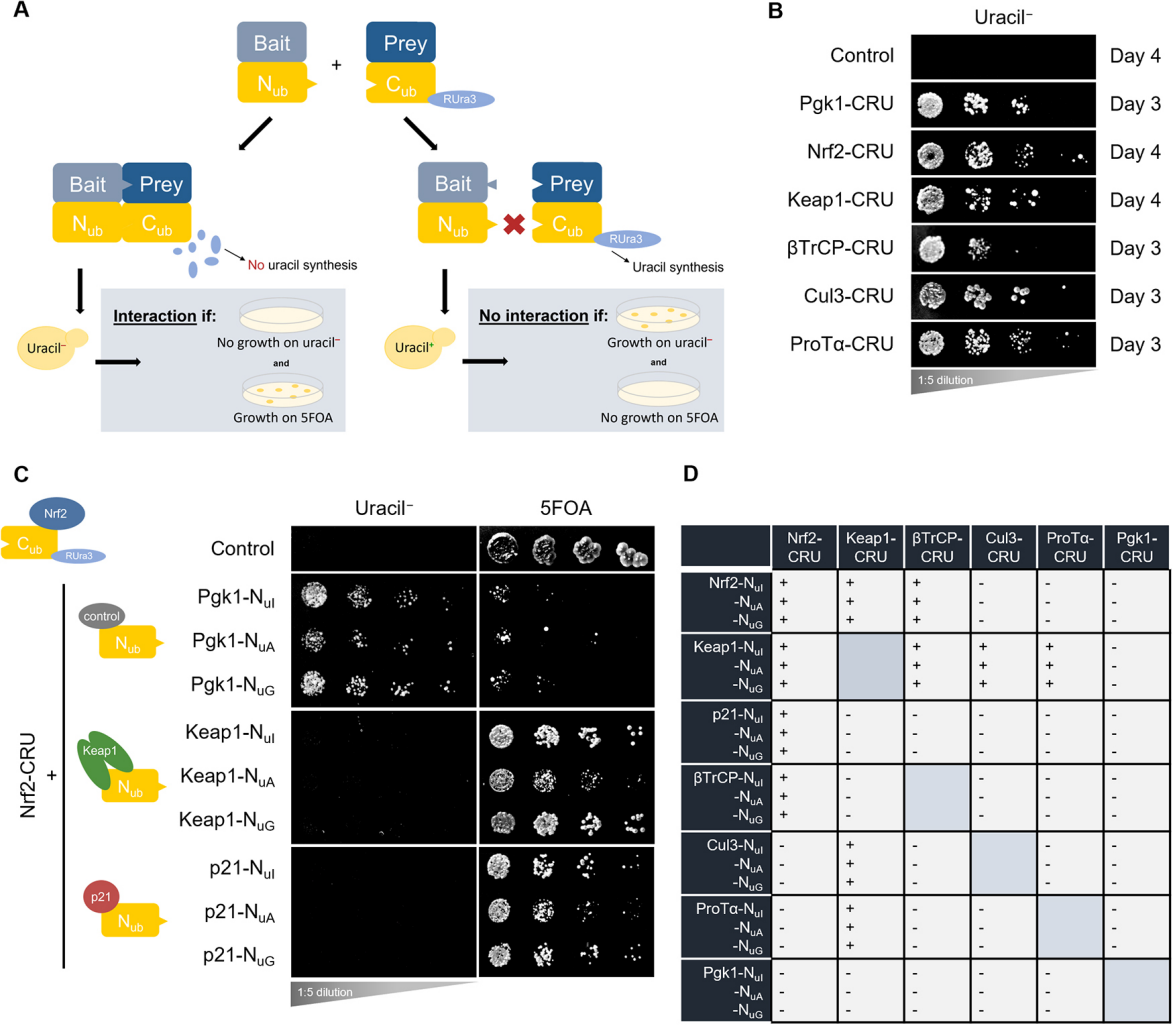
**The yeast split-ubiquitin system for studying physical Nrf2 protein-protein interactions (PPIs).** (A) Schematic representation of the split-ubiquitin system. If the bait and prey proteins interact, then the following growth conditions are met: no growth on uracil^−^ media and growth on 5FOA-containing media. (B) Confirmation of the CRU constructs showing that yeast cells expressing the CRU fusions grow on uracil^−^ plates. (C) Split-ubiquitin assays for the Nrf2-CRU+Keap1-N_ub_ and Nrf2-CRU+p21-N_ub_ combinations. Pgk1 served as a negative specificity control. Three biological replicates were performed. (D) Summary of all tested PPIs within the Nrf2 interactome as detected by the split-ubiquitin assay; ‘+’ indicates interaction and ‘−’ indicates no interaction.

We next employed the split-ubiquitin system (Johnsson and Varshavsky, 1994; Stagljar et al., 1998; Müller and Johnsson, 2008), outlined schematically in Fig. 5A, to assess the physical PPIs of Nrf2 (i.e. interactions between a ‘bait’ protein with a ‘prey’ protein). We engineered Nrf2, Keap1 and other proteins of interest fused to N_ub_, the amino-terminal half of a full-length ‘pseudo-ubiquitin’ molecule, and to C_ub_-RUra3p (CRU), the carboxy-terminal (C_ub_) half of pseudo-ubiquitin fused to a RUra3p reporter containing a degron (R) for rapid degradation by cellular ubiquitin specialized proteases (Ubps). These N_ub_ and CRU fusions were co-expressed in yeast. Nrf2 interactions could be detected by growth on the following selective growth media: (1) media lacking uracil (uracil^−^), which selects for the presence of the RUra3p reporter – an interaction between the bait and prey results in degradation of the RUra3p reporter and loss of uracil synthesis, detected by impaired growth on uracil^−^ media; (2) media containing 5-fluoroorotic (5FOA), which selects for the absence of the RUra3p reporter and associated loss of uracil synthesis (5FOA reacts with uracil to produced a toxic metabolite, 5-fluorouracil, that impairs yeast growth) – if the bait and prey interact, then the RUra3p reporter is degraded and growth is observed on 5FOA media (Fig. 5A). Different mutant alleles of N_ub_ – N_uI_, N_uA_ and N_uG_ (in order from highest to lowest affinity for C_ub_) – were used to differentiate strong interactions (e.g. in stable complexes) from weaker ones (e.g. transient interactions), where N_uI_ detects stable interactions and N_uG_ only detects transient interactions (Stagljar et al., 1998).

Fig. 5B confirmed that the CRU constructs expressed in yeast grow on agar plates lacking uracil, which is a prerequisite for the split-ubiquitin assay to work. Testing the established Keap1-Nrf2 interaction utilizing the Nrf2-CRU+Keap1 N_uI_/N_uA_/N_uG_ in yeast resulted in no growth on uracil^−^ plates but growth on 5FOA plates, which confirmed the physical PPI between Keap1 and Nrf2 (Fig. 5C, third row). This was also observed for Nrf2-CRU+p21-N_uI_/N_uA_/N_uG_, indicating a physical interaction between p21 and Nrf2 (Fig. 5C, fourth row). Phosphoglycerate kinase 1 (Pgk1), an enzyme involved in gluconeogenesis with no reported Nrf2 interactions, was used as a negative specificity control and indeed did not interact with Nrf2 in the split-ubiquitin assay (Fig. 5C, second row). Note that the low expression of the Nrf2-split-ubiquitin fusion proteins did not result in cellular toxicity compared to the high-expression plasmids used in our genetic interaction studies. We then assessed for physical interactions of all combinations of Nrf2, Keap1, p21, βTrCP, Cul3 and ProTα, summarized in Fig. 5D, with ‘+’ indicating an interaction and ‘−’ indicating no interaction, which confirmed the physical interactions between Nrf2 and Keap1, p21 and βTrCP; and between Keap1 and Nrf2, βTrCP, Cul3 and ProTα. Pgk1 again served as a negative specificity control.

### Interaction between Nrf2/Keap1 and Hsp90

**Fig. 6.**
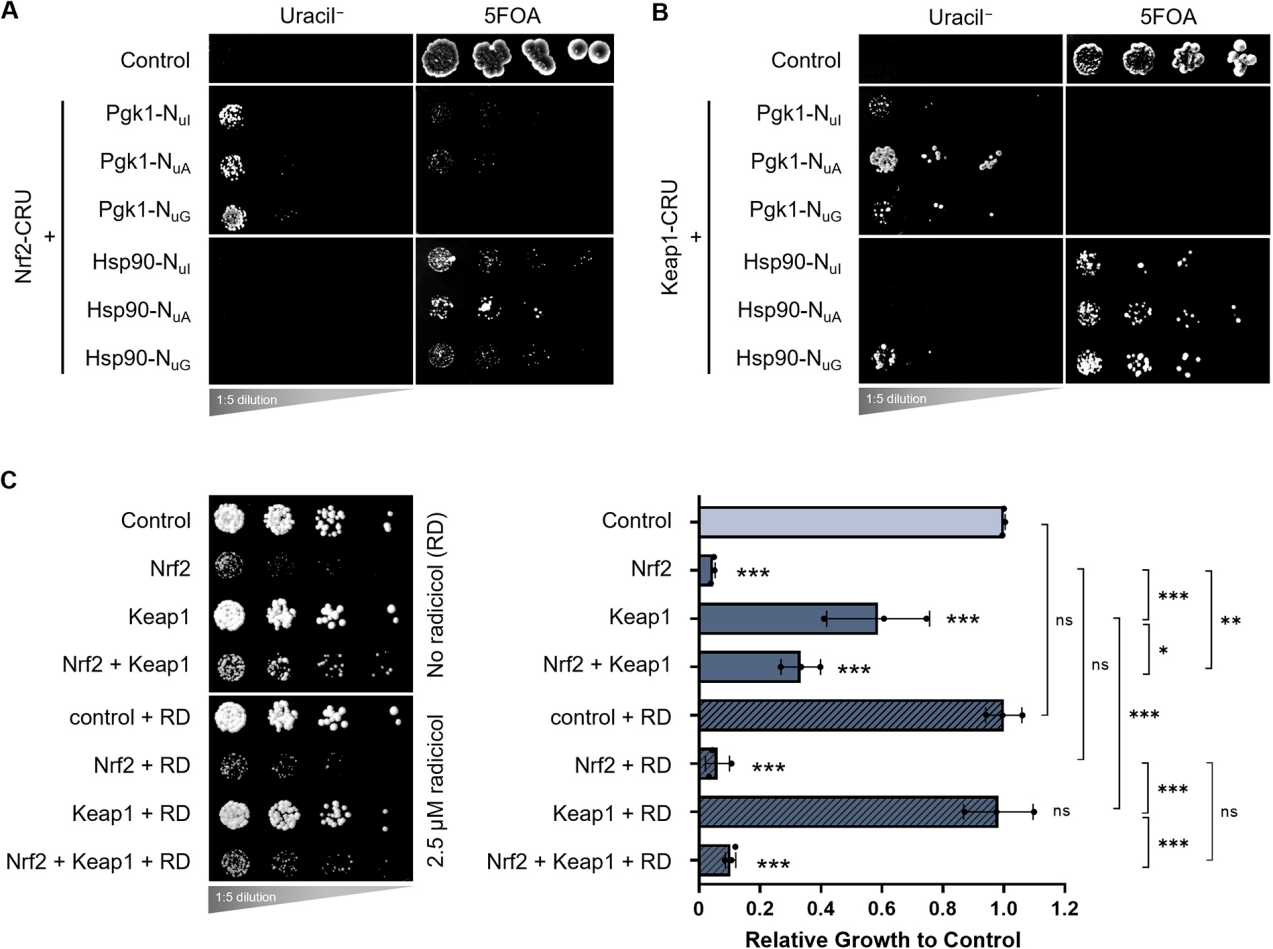
**Interactions between Nrf2 and Keap1 with Hsp90.** (A,B) Split-ubiquitin assays of yeast cells co-expressing the indicated N_ub_ and CRU fusion proteins for Nrf2 (A) and Keap1 (B), in combination with Hsp90. (C) Growth assay of yeast cells co-expressing Nrf2 and Keap1 in the absence of radicicol (RD; dimethyl sulfoxide solvent control) and presence of 2.5 µM radicicol. Means derived from three biological replicates were used during analysis. Means were analyzed using one-way ANOVA followed by Tukey's post hoc test. Data are expressed as mean±s.d. ns, not significant; *P<0.05, **P<0.01, ***P<0.001.

Because Nrf2 contains disordered regions (Itoh et al., 2004; Karunatilleke et al., 2021) and certain fragmented variants of Nrf2 form inclusions in yeast (Fig. 2C) and mammalian cells (Fig. 2D), we used the split-ubiquitin assay to probe for interactions with molecular chaperones, which help to fold, stabilize and degrade disordered and misfolded proteins (Frydman, 2001; Bukau et al., 2006). Interestingly, our split-ubiquitin data indicated that both Nrf2 and Keap1 physically interact with the molecular chaperone Hsp90 (Fig. 6A,B, respectively). Furthermore, we had previously shown that the co-expression of Keap1 and Nrf2 partially rescued Nrf2 toxicity (Fig. 3A); however, treatment with 2.5 µM radicicol, a small-molecule inhibitor of Hsp90, impaired the ability of Keap1 to rescue Nrf2 toxicity (Fig. 6C), further suggesting a genetic interaction between both Keap1 and Nrf2 with Hsp90. Of note, the interactions between Keap1 and Kelch domain-containing proteins (such as Keap1) with Hsp90 have previously been described (Taipale et al., 2012; Prince et al., 2015) but are not well explored. The interaction between Nrf2 and Hsp90, to our knowledge, has not previously been described.

**Fig. 7.**
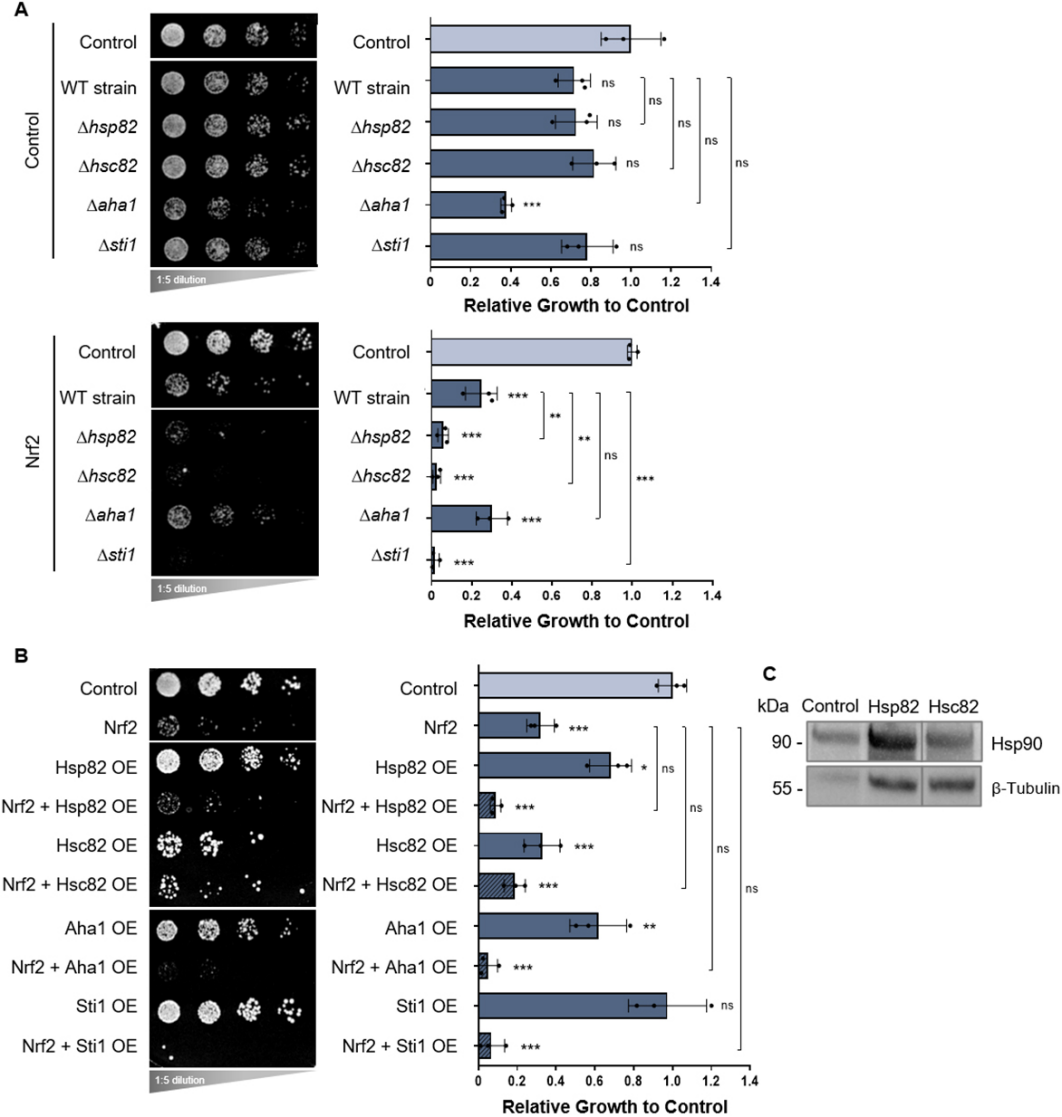
**Expression of Nrf2 in yeast Hsp90 and co-chaperone deletion strains and overexpression constructs.** (A) Growth assays of yeast cells expressing Nrf2 in deletions strains for yeast Hsp90 (Δ*hsp82* and Δ*hsc82*) and two yeast Hsp90 co-chaperones (Δ*aha1* and Δ*sti1*). (B) Growth assays of yeast cells expressing Nrf2 and overexpressing (OE) yeast Hsp90 or its co-chaperones. In A and B, means derived from three biological replicates were used during analysis. Means were analyzed using one-way ANOVA followed by Tukey's post hoc test. Data are expressed as mean±s.d. ns, not significant; *P<0.05, **P<0.01, ***P<0.001. (C) Endogenous and transformed yeast Hsp90 (Hsp82 and Hsc82) protein expression in yeast is shown by western blot analysis, with β-tubulin serving as the internal loading control.

To further investigate this interesting link between Nrf2/Keap1 and Hsp90, Nrf2 and Keap1 were expressed in deletion strains for yeast Hsp90 (Δ*hsp82* and Δ*hsc82*) and two yeast Hsp90 co-chaperones (Δ*aha1* and Δ*sti1*). Gene deletions of *HSP82*, *HSC82* or co-chaperone *STI1* exacerbated Nrf2 toxicity compared to wild-type cells, whereas the *AHA1* deletion had no effect (Fig. 7A). These deletion strains did not significantly alter Keap1 expression (Fig. S6). Nrf2 toxicity was not, however, significantly altered in cells overexpressing Hsp90 and its co-chaperones (Fig. 7B). Western blot analysis confirmed protein expression of Hsp82 and Hsc82 (both endogenous and transformed) in yeast cells for our overexpression studies (Fig. 7C). Taken together, these results indicate that Nrf2 and Hsp90 share a genetic interaction in addition to a physical interaction observed in the yeast split-ubiquitin assays.

**Fig. 8.**
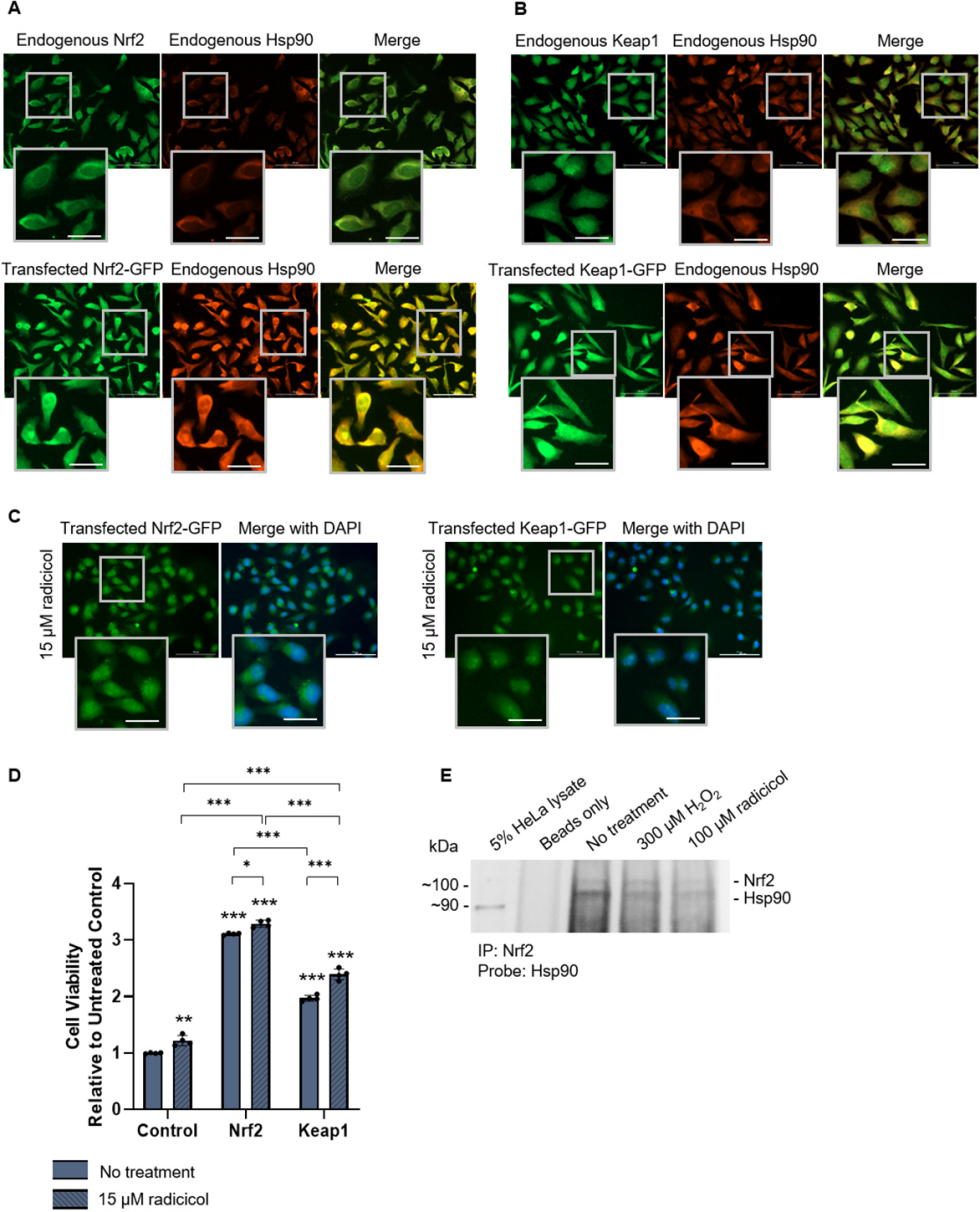
**Nrf2 and Keap1 expressed in HeLa cells with Hsp90 detection.** (A) Immunofluorescence microscopy for endogenous Nrf2 and Hsp90 (top) and fluorescence microscopy for transfected Nrf2-GFP and endogenous Hsp90 detected by immunofluorescence (bottom). (B) Immunofluorescence microscopy for endogenous Keap1 and Hsp90 (top) and fluorescence microscopy for transfected Keap1-GFP and endogenous Hsp90 detected by immunofluorescence (bottom). (C) Fluorescence microscopy of HeLa cells transfected with Nrf2-GFP and Keap1-GFP treated with 15 μM radicicol for 6 h. Scale bars: 25 μm. (D) Viability assays for HeLa cells expressing Nrf2-GFP or Keap1-GFP treated with 15 μM radicicol for 6 h. Means derived from three biological replicates were used during analysis. Means were analyzed using one-way ANOVA followed by Tukey's post hoc test. Data are expressed as mean±s.d. *P<0.05, **P<0.01, ***P<0.001. (E) Co-immunoprecipitation for endogenous Nrf2/Hsp90 complexes. Nrf2 was immunoprecipitated, followed by western blot detection for Hsp90 with subsequent probing for Nrf2. 5% HeLa lysates served as a positive control for Hsp90 but not Nrf2 as protein levels were too low for detection at a 5% lysate concentration.

Finally, we began to assess the interaction between Hsp90 and Nrf2/Keap1 in mammalian cells. Fig. 8A shows both endogenous and transfected Nrf2-GFP together with endogenous Hsp90 detected by immunofluorescence microscopy in HeLa cells. Similarly, Fig. 8B shows endogenous and transfected Keap1-GFP with endogenous Hsp90. Co-localization with Hsp90 was observed for both Nrf2 and Keap1. We then assessed the effect of Hsp90 inhibition on cells overexpressing Nrf2 and Keap1. GFP-tagged Nrf2- and Keap1-transfected HeLa cells were treated with 15 μM of the Hsp90 inhibitor radicicol for 6 h, which induced the formation of Nrf2 and Keap1 inclusions in the nucleus (Fig. 8C). Radicicol treatment also increased the viability [detected by increased ATP levels (Crouch et al., 1993)] of cells expressing Nrf2 and Keap1 compared to untreated control cells (Fig. 8D). Lastly, endogenous Nrf2/Hsp90 complexes were detected by co-immunoprecipitation (co-IP) (Fig. 8E; refer to Fig. S11 for the full blot). Nrf2 was immunoprecipitated, followed by western blot detection for Hsp90 with subsequent probing for Nrf2. Five-percent HeLa lysates served as a positive control for Hsp90 but not Nrf2, as levels of endogenous Nrf2 were too low for detection at a 5% concentration. Nrf2 and Hsp90 were detected by co-IP under both basal conditions and upon treatment with 300 µM H_2_O­_2_ and 100 µM radicicol. As expected, Nrf2 proteins levels increased with H_2_O­_2_ treatment while Hsp90 protein levels decreased with inhibition by radicicol. Thus, these results in HeLa cells support the Hsp90-Nrf2 interaction observed in yeast.

## DISCUSSION

In this study, we established the yeast *Saccharomyces cerevisiae* as a living test tube to identify and characterize genetic and physical Nrf2 interactions. Importantly, yeast does not express any close Nrf2 homolog, which allows for the advantage of minimizing interference with endogenous Nrf2 regulation as occurs in mammalian cells. Because yeast genes do not contain the antioxidant response element (ARE) required for Nrf2-mediated transcriptional activation within their promotor (Itoh et al., 1997), and yeast does not express the small MAF proteins required for Nrf2-DNA binding, Nrf2 is likely non-functional in yeast, allowing protein interactions to be examined in isolation. We, however, also acknowledge that this may also be a limitation of our yeast model because endogenous factors that may modulate Nrf2 interactions or mechanisms are not present in yeast. Importantly, in the context of Nrf2, yeast can be utilized as a ‘living test tube’ to study protein interactions, or to screen for small molecules that may alter these interactions without external interference, which may then be translated to a more biologically relevant mammalian cell or animal model.

Nrf2 contains long disordered regions (Tong et al., 2006; Karunatilleke et al., 2021) and may thus misfold easily, particularly in yeast, in which many of its interacting proteins are not expressed. This misfolding may be the cause of Nrf2 toxicity in yeast, as documented for other misfolded proteins (Outeiro and Lindquist, 2003; Duennwald, 2011; Fushimi et al., 2011). Yeast does not endogenously express proteins that, under normal conditions, lead to the rapid degradation of Nrf2 in mammalian cells (e.g. Keap1, Cul3, βTrCP), resulting in high protein expression levels of Nrf2 in yeast, which can contribute to its misfolding and ensuing toxicity. However, for HeLa cells, which do contain the mechanisms for Nrf2 activation and degradation, increased Nrf2 expression by transient transfection can enhance cellular antioxidant capacity, leading to increased cell viability (as observed), particularly in a cell culture scenario in which cells can experience artificially high levels of oxidative stress that is inherent to the system (Jagannathan et al., 2016). Here, we take advantage of Nrf2 toxicity in yeast as a tractable phenotype to study genetic Nrf2 interactions.

Along with full-length human Nrf2, we examined Nrf2 fragments to assess which region(s) of the protein contribute to its toxicity. We found that the N-terminal fragment was the most toxic in both yeast and mammalian cells and also formed protein inclusions in both systems. The Neh2 domain within the N-terminus of Nrf2 has been structurally characterized and is highly intrinsically disordered with little secondary structure (Tong et al., 2006) and, thus, a plausible main driver of Nrf2 misfolding and toxicity in yeast. Additionally, although overexpression of wild-type Nrf2, the ΔNeh2/3 variant, and C-terminal fragment in HeLa cells conferred increased cell viability – likely through increased cellular antioxidant capacity – expression of the NH_2_ fragment decreased the increased cell viability observed for wild-type Nrf2. We speculate that this may be due to the absence of the Neh1 domain, which is required for DNA/ARE-binding to activate the oxidative stress response, thereby abolishing the antioxidant advantage conferred by increased Nrf2 transcriptional activity. It is important to note that this observed advantage of Nrf2 overexpression in HeLa cells contrasts the toxicity of Nrf2 in yeast, which is likely attributed to the presence of Nrf2-related mechanisms that yeast do not possess.

Because this work focuses on characterizing a new model to study Nrf2 interactions, it is important to first confirm already known interactions to determine the validity of the model. Notably, we were able to recapitulate key Nrf2 interactions with Keap1 and other documented proteins using yeast growth assays and the split-ubiquitin system. Keap1 partially rescues wild-type Nrf2 toxicity in yeast but did not rescue toxicity for the Neh2 domain mutant variants, L30F and T80R, indicating that the impairments in the Keap1-Nrf2 interaction caused by mutations in the Keap1-binding domain [previously described by Shibata et al. (2008)] is also reflected in our yeast model. We provide evidence for other previously documented Nrf2 interactions, notably the physical interaction between Nrf2 and p21 (Chen et al., 2009), which may have strong implications in cancer. Of note, the yeast system does not detect a genetic interaction between p21 and Nrf2, possibly because yeast cells lack other cellular factors or mechanisms that characterize this interaction in mammalian cells; however, a physical interaction was observed.

Of particular interest, using yeast expression studies and the split-ubiquitin system, we document the genetic and physical interactions of Nrf2 and Keap1 with the molecular chaperone Hsp90. Chaperone perturbations by Hsp90 or co-chaperone deletions seemed to exacerbate Nrf2 toxicity in yeast, suggesting that the stability of Nrf2 is, at least in part, dependent on molecular chaperones. Future work should investigate localization patterns both in yeast and mammalian cells with such chaperone perturbations. These interactions were also observed in cultured mammalian cells, as treatment with the Hsp90-inhibitor radicicol altered Nrf2 and Keap1 localization patterns in HeLa cells; however, these data are preliminary, and more work must be done to further characterize these interactions in a mammalian system. Taipale et al. found that ubiquitin E3 ligases with Kelch domains (e.g. Keap1) interact with Hsp90 in a high-throughput study (Taipale et al., 2012), and Prince et al. have since confirmed the interaction between Keap1 and Hsp90 (Prince et al., 2015), which is further confirmed by our model. To our knowledge, the Nrf2-Hsp90 interaction detected in our studies has not previously been described. Along with the interactions observed in yeast, we provide supplemental data documenting the interaction between Nrf2 and Hsp90 by co-IP (Fig. S11), which showed modest detection of Hsp90-Nrf2 complexes in HeLa cells. Heat shock proteins and molecular chaperones, such as Hsp90, have protective roles in the refolding of proteins damaged or misfolded by cell stress and stabilizing newly synthesized proteins to ensure their correct folding (Schopf et al., 2017). Hsp90 might bind and stabilize the mostly intrinsically disordered protein Nrf2 to allow it to effectively function as a transcription factor, and may also help to stabilize Nrf2 and Keap1 to regulate their crucial interaction in the cell. Future work will determine the exact mechanisms and functional outcome of the Nrf2-Hsp90 and Keap1-Hsp90 interactions in mammalian cells.

Taken together, our results show that genetic interaction assays and the split-ubiquitin system in yeast are powerful tools to study known Nrf2 interactions and to identify previously unknown interactions. Owing to its intrinsically disordered structure and numerous binding partners, Nrf2 can, in some cases, be challenging to study in mammalian systems. Our yeast model presents a useful and effective complementary tool to explore Nrf2 regulation and function, and may serve as a platform to screen for small molecules that modulate Nrf2 interactions and activity, which has potential therapeutic value. Our work also provides evidence for the interaction of Nrf2 and Keap1 with the molecular chaperone Hsp90, and may thus indicate an important nexus between two cellular stress response pathways, i.e. the antioxidant response and the heat shock response, with possible implications in normal cellular stress regulation and cancer.

## MATERIALS AND METHODS

### Plasmids

All plasmids for yeast growth assays, fluorescence microscopy, western blots and mammalian cell expression were created using the Gateway cloning technology developed by Invitrogen (Katzen, 2007) according to the manufacturer's protocol. The yeast expression plasmids used in this study include pAG423GAL-ccdB with or without an EYFP fluorescent tag and pAG425GAL-ccdB with or without a DsRed fluorescent tag. The mammalian cell expression plasmid used was pcDNA3.1-CMV-ccdB with or without a GFP fluorescent tag. All plasmids for split-ubiquitin assays were created using traditional restriction digest and ligation-based cloning with the following plasmids: pC_ub_-RUra3, pN_uI_, pN_uA_ and pN_uG_. Note that the p21-CRU construct was not viable in *Escherichia coli* during the plasmid generation process and was excluded from the study.

### Yeast strains, culture conditions and growth assays

For assessment of relative yeast growth and protein toxicity, yeast strains derived from W303 (MAT a leu2-3,112 trp1-1 can1-100 ura3-1 ade2-1 his3-11,15) (Thomas and Rothstein, 1989) were used. Yeast deletion strains were obtained from the *Saccharomyces* Genome Deletion Project (Thomas and Rothstein, 1989). Yeast cells were transformed using the standard lithium acetate/salmon sperm carrier DNA/PEG method for the incorporation of yeast plasmids (Gietz and Schiestl, 2007). Transformed yeast cells were grown overnight in synthetic selective media to maintain the plasmid(s). Growth assays and split-ubiquitin assays were performed by spotting 5× serial dilutions of OD_600_=0.2 on selective agar plates. Following the standard for yeast growth assays, three biological replicates were performed. To induce protein expression for liquid growth assays, fluorescence microscopy and western blots, overnight cultures were washed twice with water and resuspended in media containing 2% galactose and incubated overnight. Liquid growth assays were performed using the Bioscreen C Pro Automated Microbiology Growth Curve Analysis System (Growth Curves USA).

### Spotting assay growth quantification

Quantification was carried out as described previously (Petropavlovskiy et al., 2020). In brief, yeast agar plates were imaged in black and white using the Gel Doc XR+ System (Bio-Rad). Images were pre-processed using Image Lab Software (Bio-Rad) to remove color and background data. Images were then imported into ImageJ (National Institutes of Health), and white pixel count was measured and summed for dilutions 1-3 for each condition. Data were quantified relative to the empty vector control on the same respective plate and imported into Prism 8 (GraphPad Software), for generation of scatter dot plots with bars. Three biological replicates were analyzed. Statistical analysis was performed as described at the end of this section.

### Yeast fluorescence microscopy

For assessment of fluorescently tagged protein expression and localization, yeast strains derived from BY 4741 (MAT α his3Δ1 leu2Δ0 lys2Δ0 ura3Δ0) (Brachmann et al., 1998) were used. Yeast expression plasmids were tagged with either YFP or DsRed. Cells were transferred to a glass microscope slide and coverslip and imaged using either the Olympus BX-51 Bright Field/Fluorescence Microscope at 60×, captured using an equipped CCD camera (Spot Pursuit), or the Cytation 5 Cell Imaging Multi-Mode Reader (BioTek) at 20×, captured using Gen5 Software (BioTek).

### Electrophoresis and western blot analysis

Proteins were extracted from yeast cells using the alkaline lysis method (Kushnirov, 2000). Lysate (30 µl) was resolved on an SDS-PAGE gel. The membrane was blocked with 5% bovine serum albumin (BSA) in PBST [1× phosphate-buffered saline (PBS), 0.1% Tween-20] and incubated with primary antibody overnight (refer to Table S1). The membrane was incubated with a horseradish peroxidase (HRP)-conjugated secondary antibody for 1 h at room temperature, either anti-rabbit (Abcam, ab6721), anti-mouse (Abcam, ab6728) or anti-rat (Abcam, ab97057), as required at a concentration of 1:1500. Western blots were visualized using the Clarity Western ECL Substrate kit (Bio-Rad, 1705061), and images were taken using the ChemiDoc Imaging System (Bio-Rad). Antibodies were tested on three biological replicates to ensure specificity (Fig. S10).

### Mammalian cell culture conditions and transfections

The HeLa and HEK293 cell lines were maintained in Dulbecco's modified Eagle medium (DMEM; Gibco, 41966-029), supplemented with 10% fetal bovine serum (Wisent, 080-150) and 1× penicillin-streptomycin (Corning, 30-001-CI). Cell lines were not recently authenticated and tested for contamination. Cells were cultured at 37°C with 5% CO_2_. For transfections, cells were seeded in a six-well plate at 1.0×10^6^ cells per well and grown to ∼80% confluency. Cells were transfected using Lipofectamine LTX with PLUS Reagent (Thermo Fisher Scientific, A12621) according to the manufacturer's protocol in Opti-MEM I Reduced Serum Medium (Gibco, 31985-062). Transfected cells were incubated at 37°C for 6 h followed by incubation in DMEM for 18 h at 37°C. Cells were then split into the appropriate plates for subsequent experiments.

### Immunofluorescence microscopy

Transfected HeLa cells were seeded onto 15 mm circular glass coverslips (Matsunami, C015001) in a 12-well plate at 1×10^5^ cells per well to ensure ∼80% confluency the following day. Cells were fixed with 4% paraformaldehyde, permeabilized with 0.1% Triton X-100 in PBS, blocked with 20% goat head serum in PBB (0.5% BSA in PBS) and incubated with one of the following primary antibodies overnight at 4°C at a concentration of 1:100: mouse anti-Nrf2 (Abcam, ab62352), mouse anti-Keap1 (Proteintech, 10503-2-AP) or rabbit anti-Hsp90 (Proteintech, 13171-1-AP). Coverslips were incubated with the following Alexa Fluor 680-conjugated secondary antibody for 1 h at room temperature at a concentration of 1:1500: goat anti-mouse (Thermo Fisher Scientific, A-11094) or goat anti-rabbit (Thermo Fisher Scientific, A11036). Coverslips were then mounted onto glass microscope slides with SlowFade Gold Antifade Mountant with 4′,6-diamidino-2-phenylindole (DAPI; Thermo Fisher Scientific, S36938) and cured at room temperature for 24 h. Cells were imaged using the Cytation 5 Cell Imaging Multi-Mode Reader (BioTek) using a 20× objective lens and captured using Gen5 Software (BioTek).

### Cell viability assays

Transfected HeLa or HEK293 cells were seeded into 96-well solid white microplates (Greiner, M0187-32EA) at 4×10^4^ cells per well and incubated for 16 h. Following treatment, cell viability was assessed using the CellTiter-Glo 2.0 Cell Viability Assay (Promega, G9242) according to the manufacturer's protocol. Luminescence was measured using the Cytation 5 Cell Imaging Multi-Mode Reader (BioTek).

### Co-IP

HeLa cells were seeded into T75 flasks (Nunc, 156499) at 1.5×10^6^ and incubated until 85-90% confluency was reached. Following treatment, cells were scraped in ice-cold RIPA buffer [150 mM NaCl, 50 mM Tris-HCl, pH 7.5, 1% Triton X-100, 0.5% sodium deoxycholate and 0.1% SDS, supplemented with Halt Protease and Phosphatase Inhibitor Cocktail (Thermo Fisher, 78440)] and incubated on ice for 10 min. For further lysis, lysates underwent two sequential freeze/thaw cycles of −80°C and 37°C and centrifuged at 17,000 ***g*** at 4°C for 10 min. Co-immunoprecipitation was performed using the SureBeads Protein A Magnetic Beads (Bio-Rad, 161-4013) according to the manufacturer's protocol, with modifications where specified. Nrf2 was immunoprecipitated using an anti-rabbit primary antibody to Nrf2 (Abcam, ab62352) for 20 min at room temperature, and lysates were incubated with the Nrf2-magnetic bead complexes overnight at 4°C. Samples were resolved on an SDS-PAGE gel, and the membrane was blocked with 5% BSA in PBST. The membrane was incubated with anti-mouse primary antibody to Hsp90 (Abcam, ab13492) overnight at 4°C at a concentration of 1:1000, followed by incubation with an HRP-conjugated anti-mouse secondary antibody (Abcam, ab6728) for 1 h at room temperature and then imaged. The membrane was then incubated with anti-rabbit primary antibody to Nrf2 (Abcam, ab62352) overnight at 4°C at a concentration of 1:1000, followed by incubation with an HRP-conjugated anti-rabbit secondary antibody (Abcam, ab62352) for 1 h at room temperature and then imaged. Western blots were visualized using the Clarity Western ECL Substrate kit (Bio-Rad, 1705061), and images were taken using the ChemiDoc Imaging System (Bio-Rad).

### Statistical analysis

Statistical analyses were conducted using Prism 8 (GraphPad Software). A sample size of *n*=3 (or more) biological replicates was used following the standard used in yeast expression studies. Statistical significance was obtained by performing a one-way ANOVA with Tukey post hoc for comparison between groups, or two-tailed unpaired Student's *t*-test for comparison between two groups (with a minimum of three biological replicates). Error bars represent s.d. *P*-values less than 0.05 were considered statistically significant. Shapiro–Wilk tests were performed for all data sets to ensure normality.

## Supplementary Material

Supplementary information
