# Supplementary material for: A novel yeast model detects Nrf2 and Keap1 interactions with Hsp90
Source: Dis Model Mech. 2022 Apr 13;15(4):dmm049235. doi: 10.1242/dmm.049258 (PMC9016900; doi:10.1242/dmm.049258)
Supplement: Supplementary information [file dmm-15-049258-s1.pdf]

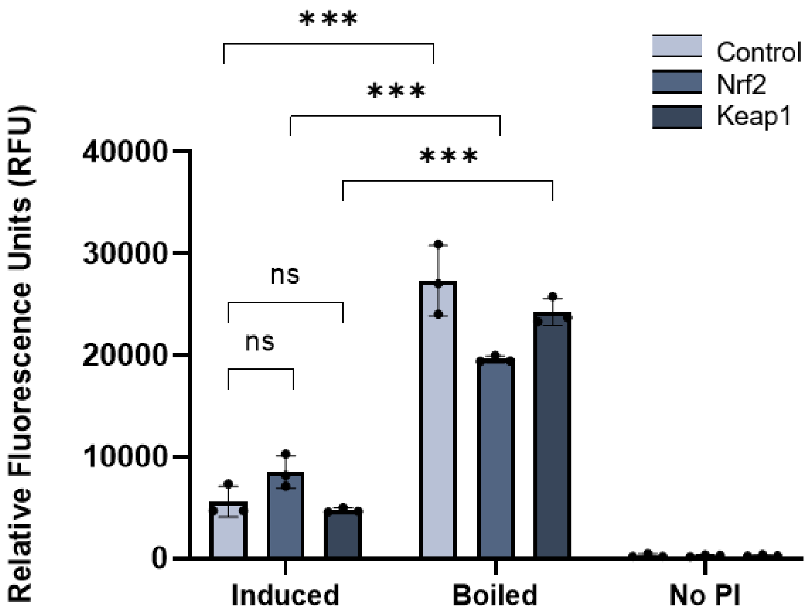

Fig. S1. Propidium iodide assay for Nrf2 and Keap1 expressed in yeast. Induced expression of Nrf2 and Keap1 in yeast was monitored by propidium iodide (PI) assays. Boiled cells served as a positive control for cell death. Means derived from three biological replicates were used during analysis. Means were analyzed using one-way ANOVA followed by Tukey's post hoc test. Data are expressed as mean ± S.D. $p<0.05$ was considered statistically significant; *$p<0.05$, **$p<0.01$, ***$p<0.001$.

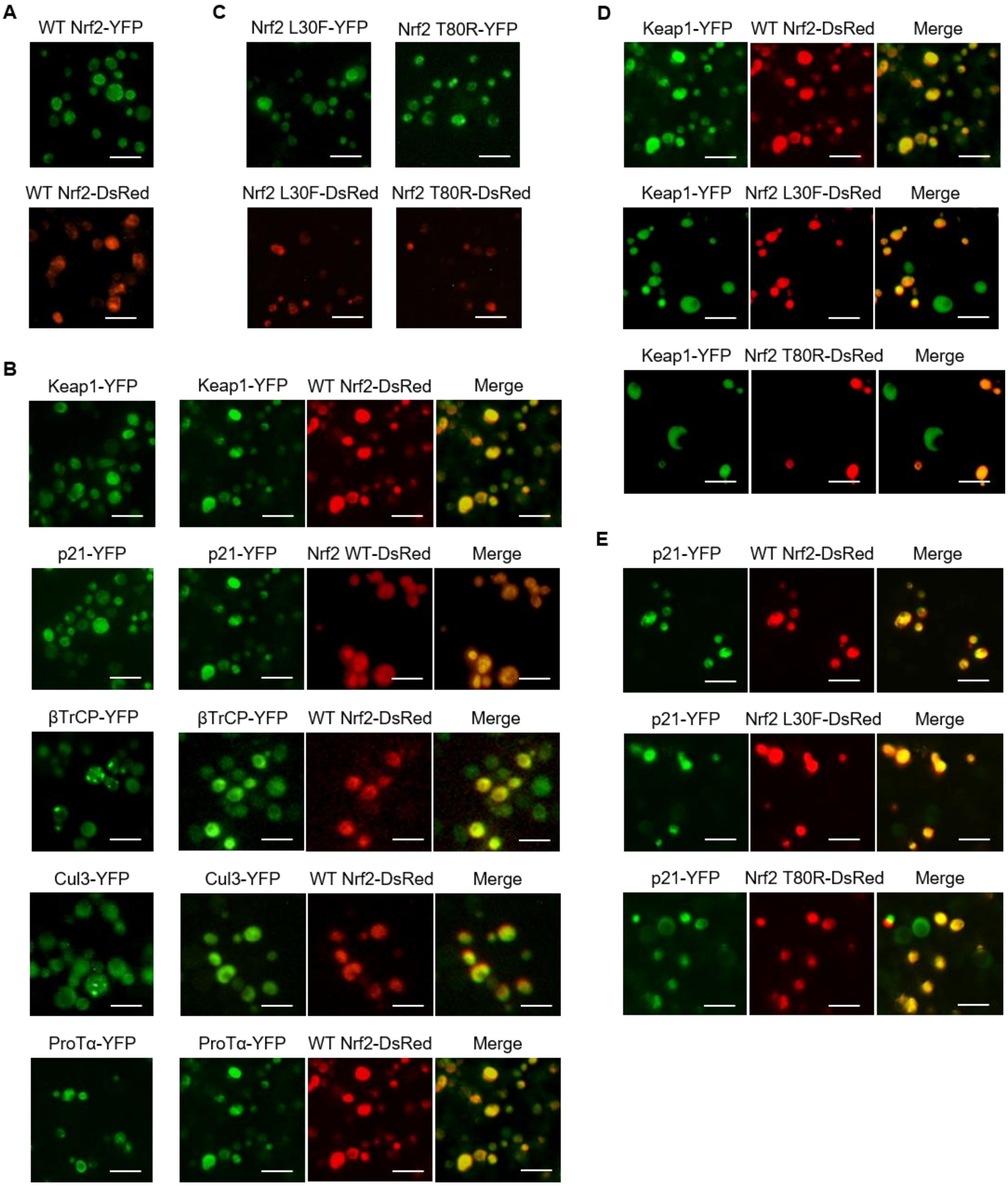
A
WT Nrf2-YFP
WT Nrf2-DsRed
C
Nrf2 L30F-YFP
Nrf2 T80R-YFP
Nrf2 L30F-DsRed
Nrf2 T80R-DsRed
D
Keap1-YFP
WT Nrf2-DsRed
Merge
Keap1-YFP
Nrf2 L30F-DsRed
Merge
Keap1-YFP
Nrf2 T80R-DsRed
Merge
B
Keap1-YFP
Keap1-YFP
WT Nrf2-DsRed
Merge
p21-YFP
p21-YFP
Nrf2 WT-DsRed
Merge
βTrCP-YFP
βTrCP-YFP
WT Nrf2-DsRed
Merge
Cul3-YFP
Cul3-YFP
WT Nrf2-DsRed
Merge
ProTα-YFP
ProTα-YFP
WT Nrf2-DsRed
Merge
E
p21-YFP
WT Nrf2-DsRed
Merge
p21-YFP
Nrf2 L30F-DsRed
Merge
p21-YFP
Nrf2 T80R-DsRed
Merge

Fig. S2. Fluorescence microscopy for all proteins of interest expressed in yeast. **(A)** Fluorescence microscopy of yeast cells expressing YFP- and DsRed-tagged wild-type Nrf2. **(B)** Fluorescence microscopy of yeast cells expressing YFP-tagged Keap1, p21, βTrCP, Cul3, and ProTα alone and co-expressed with Nrf2-DsRed. **(C)** Fluorescence microscopy of yeast cells expressing YFP- and DsRed-tagged Nrf2 mutant variants, L30F and T80R. **(D)** Fluorescence microscopy of yeast cells expressing Keap1-YFP co-expressed with DsRed-tagged wild-type or mutant Nrf2. **(E)** Fluorescence microscopy of yeast cells expressing p21-YFP co-expressed with DsRed-tagged wild-type or mutant Nrf2. All scale bars correspond to 10 µm.

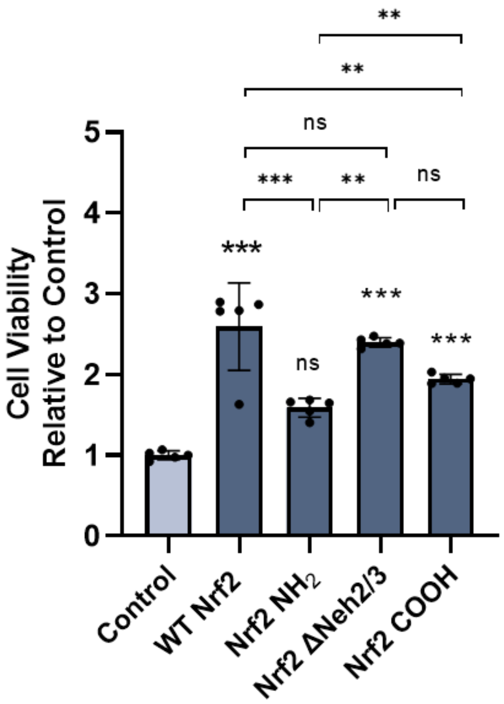

Fig. S3. Cell viability assay for wild-type Nrf2 and its fragmented variants expressed in HEK293 cells. Results recapitulate those observed in HeLa cells (Figure 2E). Means derived from five biological replicates were used during analysis. Means were analyzed using one-way ANOVA followed by Tukey's post hoc test. Data are expressed as mean ± S.D. $p<0.05$ was considered statistically significant; *$p<0.05$, **$p<0.01$, ***$p<0.001$.

Disease Models & Mechanisms • Supplementary information

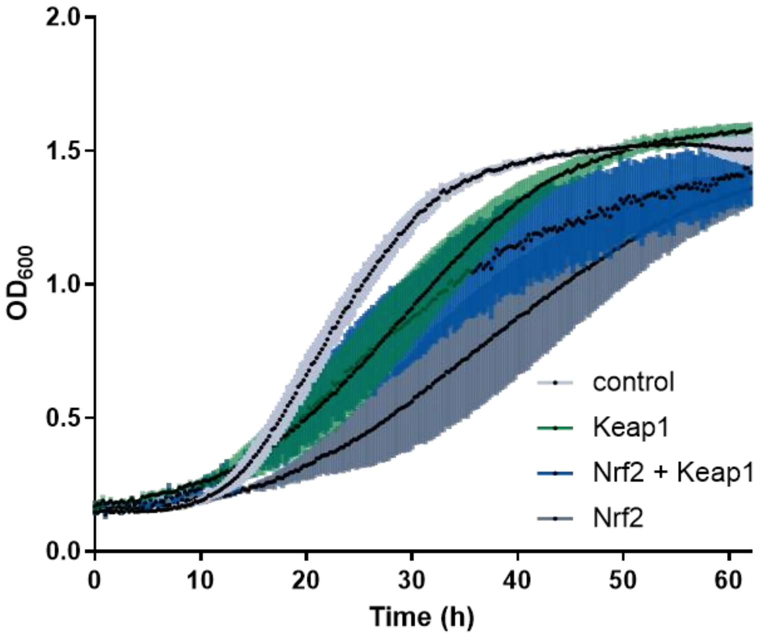

Fig. S4. Liquid growth curve for the co-expression of Nrf2 and Keap1 in yeast. Results recapitulate those observed on solid growth media. Means derived from three biological replicates were used during analysis. Data are expressed as mean (shown in black) ± S.D. (shown in colour).

Disease Models & Mechanisms • Supplementary information

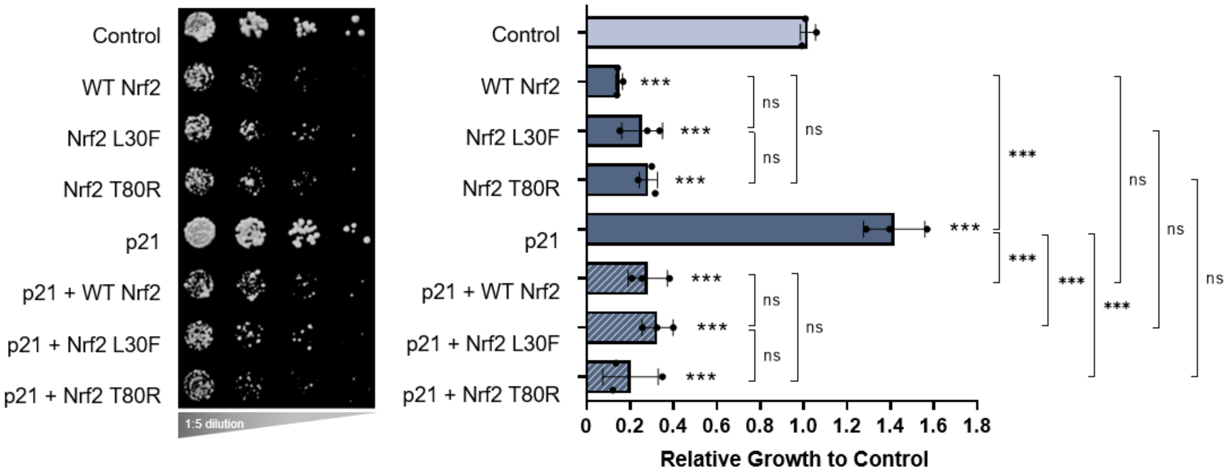

Fig. S5. p21 co-expressed with Nrf2 mutant variants in yeast. Growth assays were performed for yeast cells co-expressing wild-type Nrf2 or its mutant variants L30F and T80R with p21. Means derived from three biological replicates were used during analysis. Means were analyzed using one-way ANOVA followed by Tukey's post hoc test. Data are expressed as mean ± S.D. $p<0.05$ was considered statistically significant; *$p<0.05$, **$p<0.01$, ***$p<0.001$.

Disease Models & Mechanisms • Supplementary information

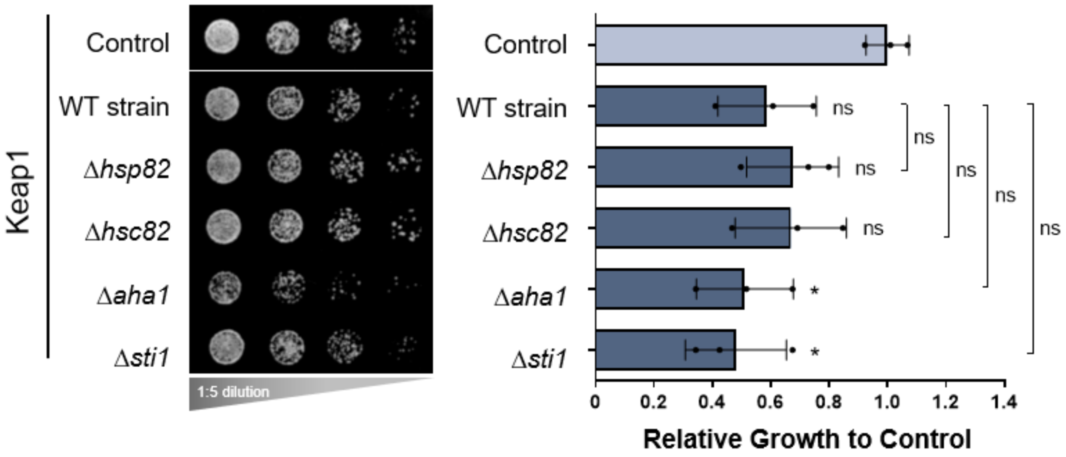

Fig. S6. Keap1 expressed in yeast Hsp90 deletion strains. Growth assays of yeast cells expressing Keap1 in deletions strains for yeast Hsp90 (Δ*hsp82* and Δ*hsc82*) and its co-chaperones (Δ*aha1* and Δ*sti1*). Means derived from three biological replicates were used during analysis. Means were analyzed using one-way ANOVA followed by Tukey's post hoc test. Data are expressed as mean ± S.D. $p<0.05$ was considered statistically significant; *$p<0.05$, **$p<0.01$, ***$p<0.001$.

Disease Models & Mechanisms • Supplementary information

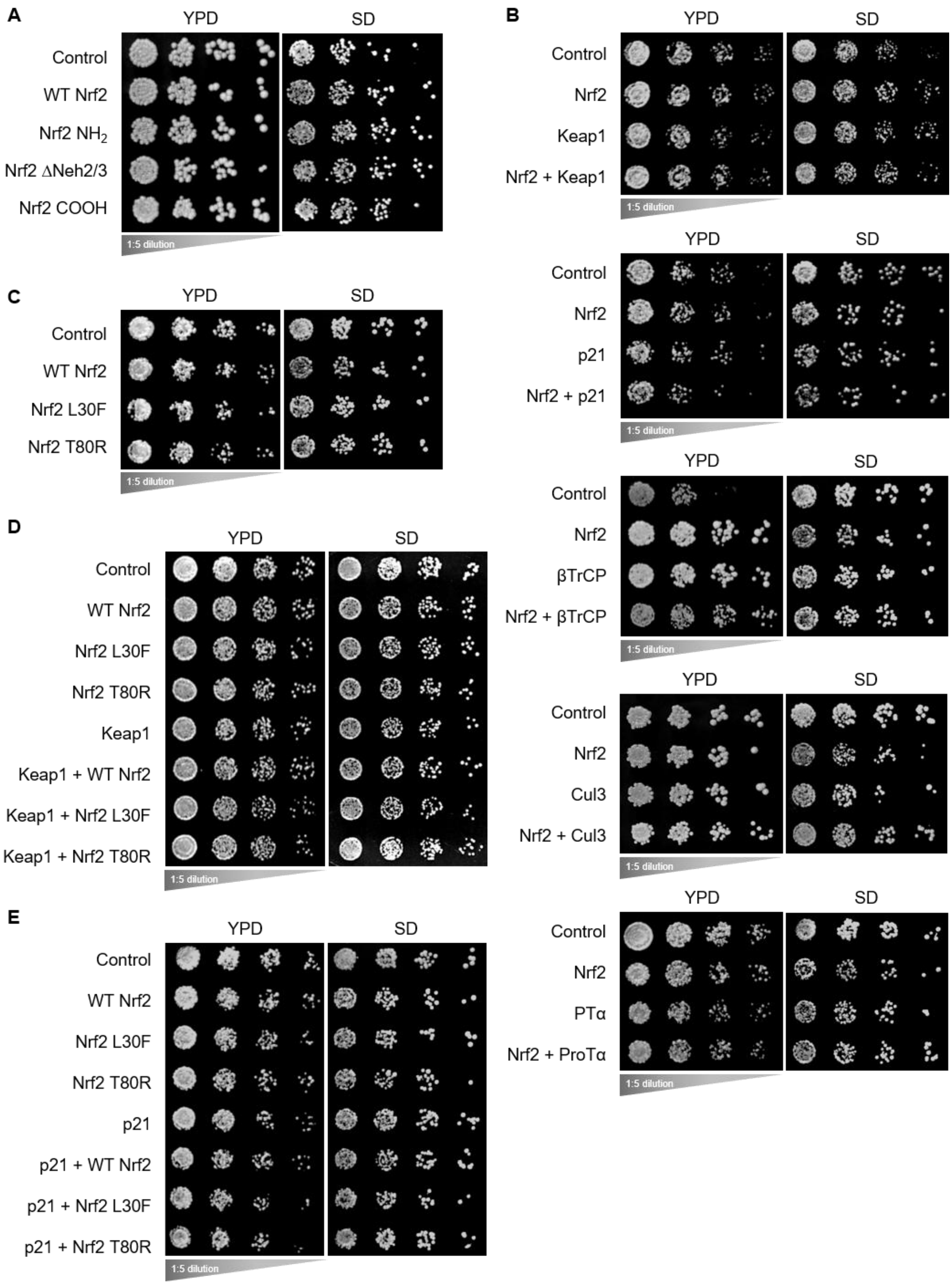

Fig. S7. (A-E) Control plates for yeast growth assay interaction studies. Yeast extract-peptone-dextrose (YPD) and selective dextrose (SD) control plates are shown.

Disease Models & Mechanisms • Supplementary information

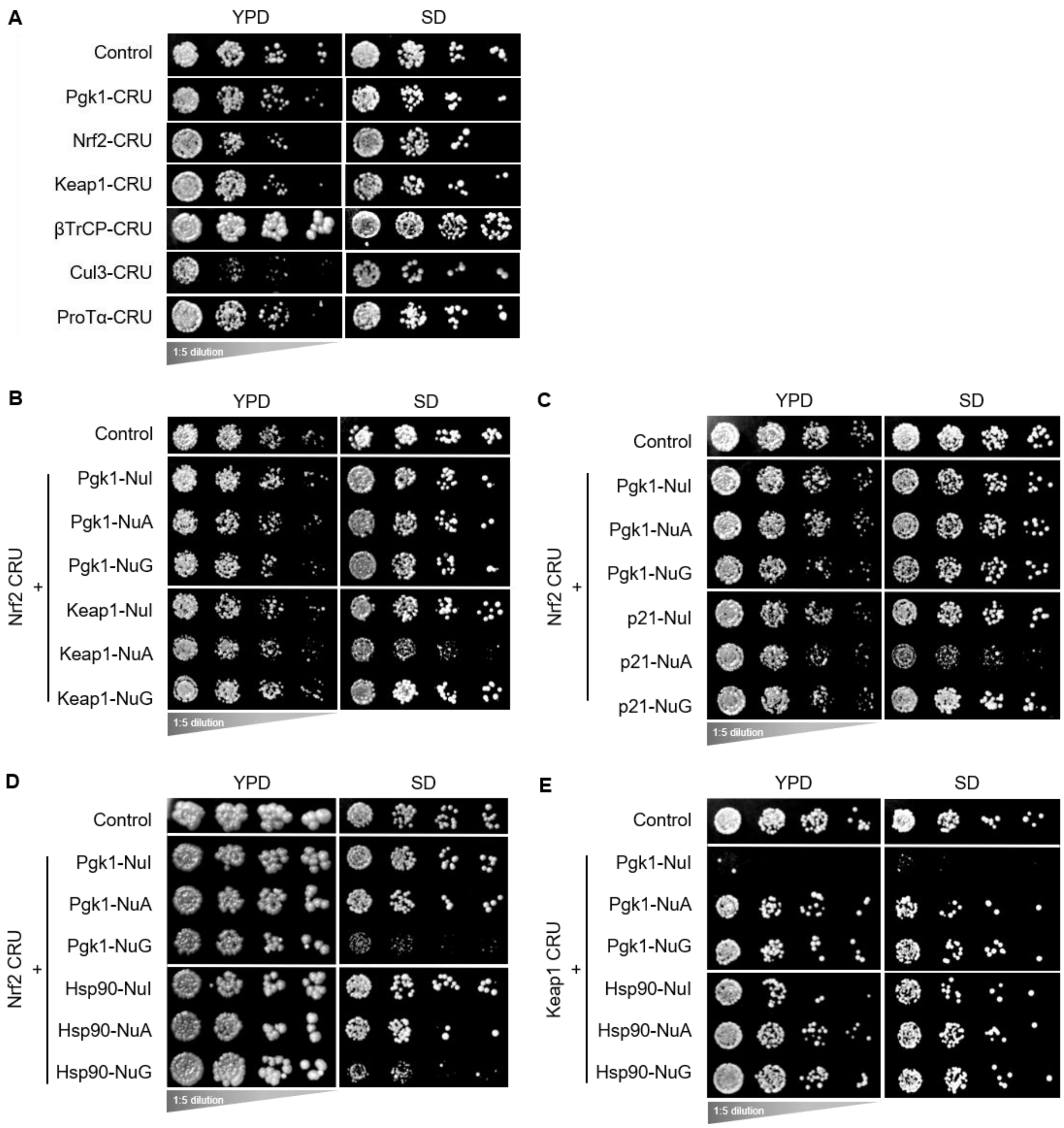

Fig. S8. (A-E) Control plates for yeast split-ubiquitin interaction studies. Yeast extract-peptone-dextrose (YPD) and selective dextrose (SD) plates are shown.

Disease Models & Mechanisms • Supplementary information

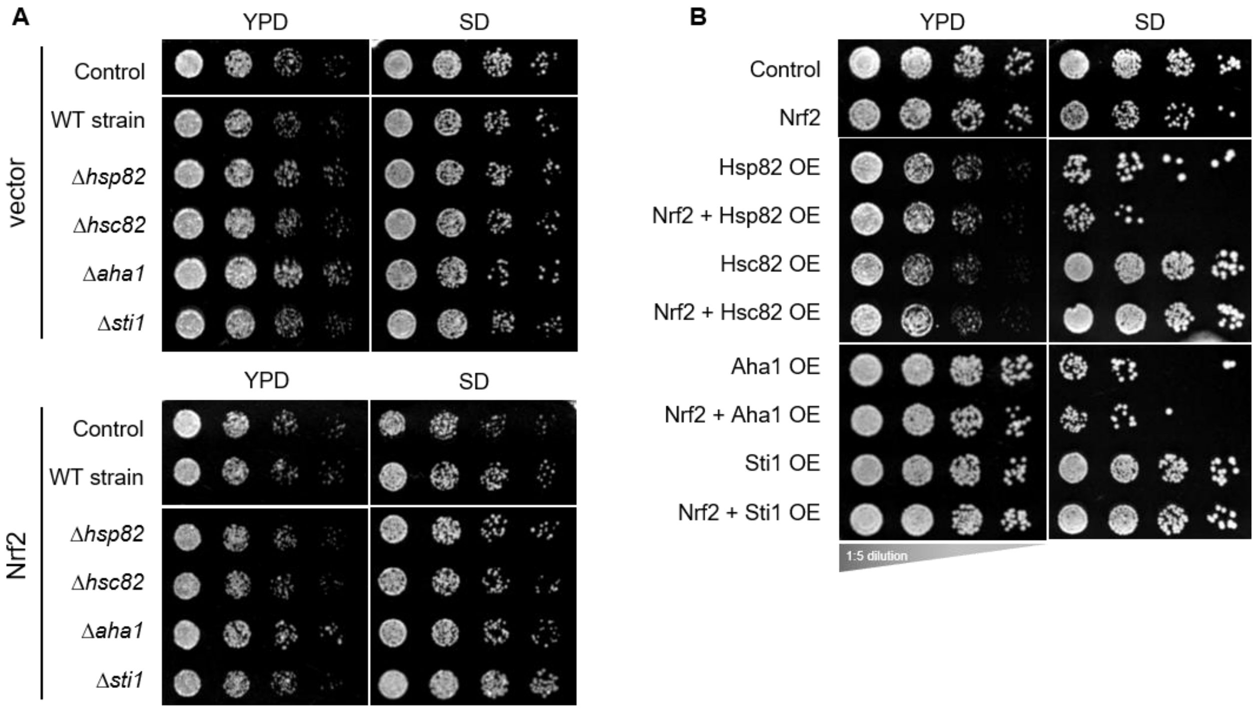

Fig. S9. (A-B) Control plates for yeast Hsp90 gene deletion and overexpression studies.

Yeast extract-peptone-dextrose (YPD) and selective dextrose (SD) plates are shown.

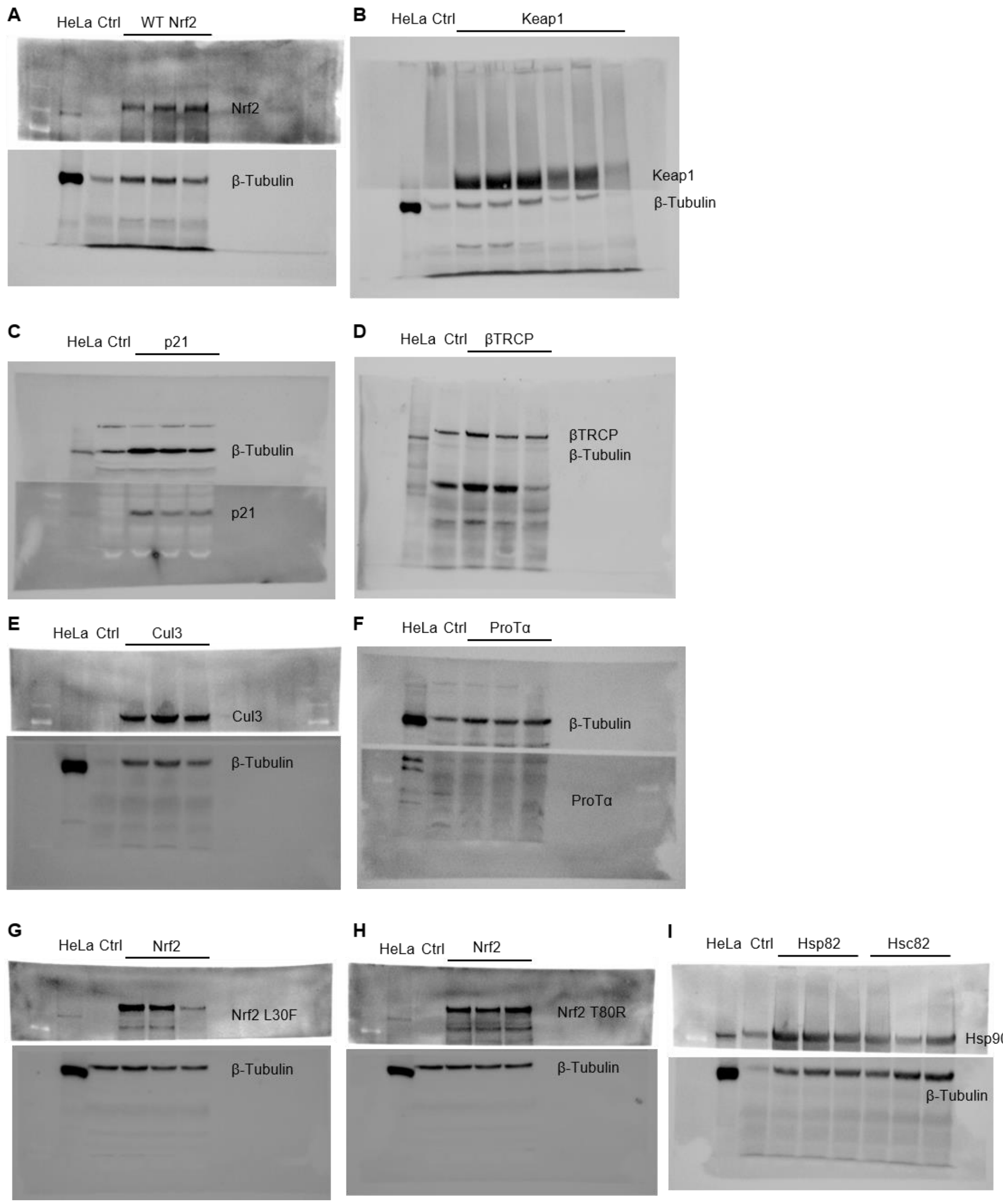

Fig. S10. (A-I) Raw western blots for all proteins of interest expressed in yeast. β-tubulin served as the internal loading control. Three or more biological replicates are shown, with HeLa cell lysates serving as a positive control and an empty vector yeast control serving as a negative control (except for proteins that are endogenously expressed in yeast).

Disease Models & Mechanisms • Supplementary information

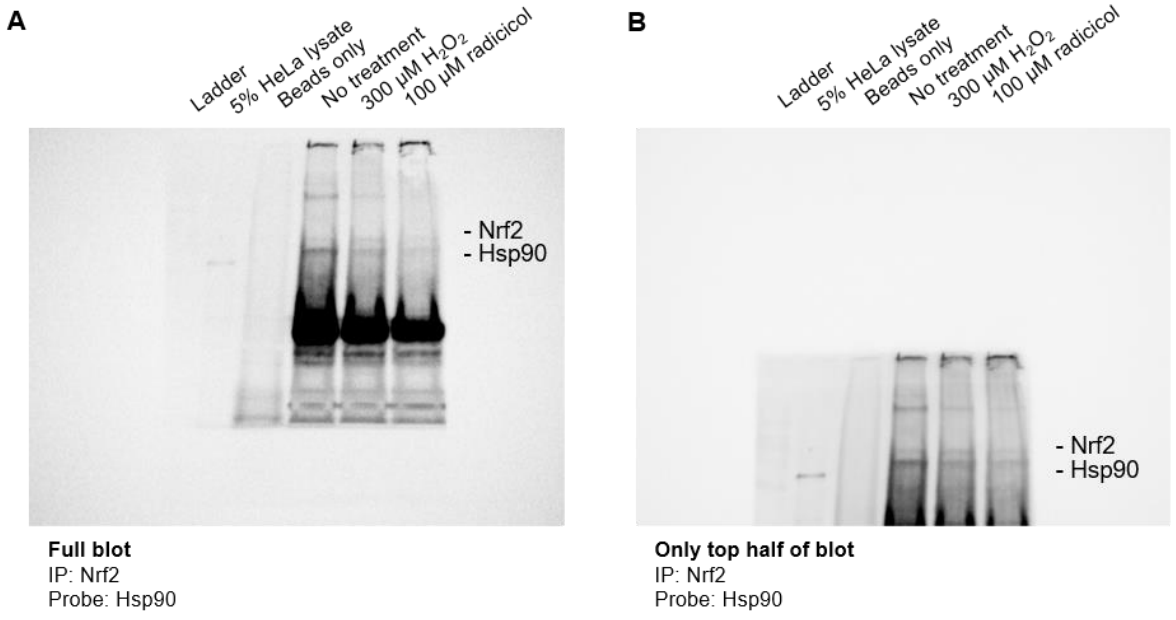

Fig. S11. Co-immunoprecipitation of Nrf2 and Hsp90 detected by western blot analysis. The same western blot is imaged to show **(A)** the full blot, and **(B)** only the top half of the blot, imaged accordingly to reduce underexposure of the signal caused by strong background signals.

Table S1. Antibodies used for western blot analyses

Antigen	Supplier	Product Code	Species	Dilution
Nrf2	Abcam	ab62352	Rabbit	1:1000
Keap1	Proteintech	10503-2-AP	Rabbit	1:1000
p21/p21$^{CiP/Waf}$	Cell Signaling	2947S	Rabbit	1:1000
βTrCP	Santa Cruz	sc-390629	Mouse	1:50
Cul3	Bethyl Laboratories	A301-109A	Rabbit	1:2000
ProTα/PTMA	(1) Invitrogen	PA5-75828	Rabbit	1:500
	(2) LifeSpan BioSciences	LS-C162288	Rabbit	1:1000
Hsp90	Abcam	ab13492	Mouse	1:1000
β-Tubulin	Abcam	ab6160	Rat	1:5000
